# METTL3-Mediated m^6^A Methylation Regulates Muscle Stem Cells and Muscle Regeneration by Notch Signaling Pathway

**DOI:** 10.1155/2021/9955691

**Published:** 2021-05-14

**Authors:** Yu Liang, Hui Han, Qiuchan Xiong, Chunlong Yang, Lu Wang, Jieyi Ma, Shuibin Lin, Yi-Zhou Jiang

**Affiliations:** ^1^Center for Translational Medicine, Institute of Precision Medicine, The First Affiliated Hospital, Sun Yat-sen University, Guangzhou 510080, China; ^2^Institute for Advanced Study, Shenzhen University, Shenzhen 518057, China; ^3^State Key Laboratory of Oral Diseases & National Clinical Research Center for Oral Diseases, West China Hospital of Stomatology, Sichuan University, Chengdu 610065, China

## Abstract

The Pax7+ muscle stem cells (MuSCs) are essential for skeletal muscle homeostasis and muscle regeneration upon injury, while the molecular mechanisms underlying muscle stem cell fate determination and muscle regeneration are still not fully understood. N6-methyladenosine (m^6^A) RNA modification is catalyzed by METTL3 and plays important functions in posttranscriptional gene expression regulation and various biological processes. Here, we generated muscle stem cell-specific METTL3 conditional knockout mouse model and revealed that METTL3 knockout in muscle stem cells significantly inhibits the proliferation of muscle stem cells and blocks the muscle regeneration after injury. Moreover, knockin of METTL3 in muscle stem cells promotes the muscle stem cell proliferation and muscle regeneration *in vivo*. Mechanistically, METTL3-m^6^A-YTHDF1 axis regulates the mRNA translation of Notch signaling pathway. Our data demonstrated the important *in vivo* physiological function of METTL3-mediated m^6^A modification in muscle stem cells and muscle regeneration, providing molecular basis for the therapy of stem cell-related muscle diseases.

## 1. Introduction

The skeletal muscle stem cells (MuSCs, satellite cells) are the stem cells located under the basal layer of mature muscle fibers and are necessary for normal muscle growth and regeneration. In resting adult muscle, MuSCs are mainly quiescent, but the MuSCs will be activated under pathological stimuli for muscle repair and tissue homeostasis [1, 2]. When muscle injury occurs, the Pax7+ MuSCs become activated and proliferate as progenitor cell myoblasts, which then differentiate into myotubes and further mature as functional muscles. The process of MuSC differentiation and fusion is mediated by several transcription factors termed the myogenic regulatory factors (MRFs) including myoblast determination protein (MyoD), myosin, and myogenin (Myog) [3]. The mechanisms controlling the muscle stem cells and muscle regeneration are complicated and still not fully understood.

Posttranscriptional modifications on RNAs, particularly the most abundant mRNA modification, N6-methyladenosine(m^6^A), have pronounced effects on mRNA stability, splicing, and translation [4–8]. The m^6^A modification requires a defined set of “writer” proteins including the active methyltransferase METTL3 and its cofactor METTL14, to deposit the methyl groups on mRNAs [9, 10]. On the other hand, the demethylases FTO and ALKBH5 catalyze the demethylation of m^6^A on mRNAs. Therefore, the m^6^A modification is reversible and dynamically regulated at different biological processes. Different reader proteins including the YTHDF, YTHDC, and IGFBP family proteins specifically recognize the m^6^A modifications on mRNAs and facilitate the stability, processing, transport, and translation of mRNAs. Therefore, the m^6^A modification plays important functions in posttranscriptional gene expression regulation.

Given the important function of m^6^A mRNA modification in gene expression, emerging evidence revealed the critical role of m^6^A in different biological processes. Recent studies revealed that METTL3-mediated m^6^A modification is essential for stem cells self-renewal and appropriate differentiation [11]. Interestingly, in myoblasts, METTL3 can promote MyoD mRNA maintenance in proliferative myoblasts for skeletal muscle differentiation [12]. Another study reported that METTL3 regulates the myoblast transition from proliferation to differentiation [13]. And depletion of FTO in myoblasts led to impaired skeletal muscle development [14]. These data suggested that the m^6^A modification could mediate the muscle progenitor cell proliferation and differentiation *in vitro*; however, the function and mechanism of METTL3-mediated m^6^A modification in regulation of muscle stem cells and muscle regeneration in vivo are inconclusive.

In this study, we established the muscle stem cell-specific METTL3 conditional knockout and conditional knockin mouse models and the muscle regeneration model to study the functions of METTL3 in regulation of muscle stem cells and muscle regeneration. We also identified Notch signaling pathway as important downstream target of METTL3 in the muscle stem cells. Our data uncovered novel posttranscriptional modification mechanism regulating muscle stem cells and muscle regeneration, providing molecular basis for the therapy of stem cell-related muscle diseases.

## 2. Materials and Methods

### 2.1. Generation of Muscle Stem Cell-Specific METTL3 Conditional Knockout and Knockin Mice

The METTL3^flox/wt^ mouse strain and the METTL3^ki/wt^ mouse strain were generated with CRISPR/Cas9 technology by Biocytogen Jiangsu Co., Ltd. Pax7CreERT2 mouse was purchased from The Jackson Laboratory. The METTL3^flox/wt^ mouse was crossed with Pax7CreERT2 mouse to generate the muscle stem cell-specific METTL3 conditional knockout mice (Pax7CreERT2; METTL3^flox/flox^, cKO) and the corresponding control mice. While METTL3^ki/wt^ mouse was crossed with Pax7CreERT2 mouse to generate the muscle stem cell-specific METTL3 conditional knockin mice (Pax7CreERT2; METTL3^ki/wt^, cKI) and the corresponding control mice. Mice were genotyped by PCR using standard protocols.

### 2.2. Muscle Regeneration Procedure

Tamoxifen (Sigma) was administered intraperitoneally at 1 mg per 20 g body weight per injection. Cardiotoxin (CTX 0.06 mg/ml, Sigma) was injected into tibialis anterior (TA) muscles in a volume of 50 *μ*l. All animal procedures were approved by the Laboratory Animal Center of Sun Yat-sen University and in accordance with the National Institutes of Health guide for the care and use of laboratory animals (NIH Publications No. 8023, revised 1978).

### 2.3. Immunofluorescence

The muscles were dipped into OCT (SAKUR) and immediately frozen in liquid nitrogen. 5 *μ*m cryosections were collected on adhesive slides (CITOGLAS) and fixed in 4% paraformaldehyde (PFA) for 10 min. Blocking was performed in blocking buffer (20% donkey serum,1% BSA,0.2% Triton X-100 in 1x PBS) for 30 min at room temperature. Appropriate primary antibody diluted in blocking buffer was used to incubate the slides overnight at 4°C. After three times washed in 1x PBS, the secondary antibody diluted in blocking buffer was added and incubated for 50 min at room temperature. Nuclei were visualized by DAPI stain. Mouse anti-Pax7 (1 : 20, DSHB), mouse anti-myogenin (1 : 20, DSHB), mouse anti-myosin (1 : 20, DSHB), mouse secondary antibodies (1 : 200, Thermo Fisher), rabbit anti-ki67 (1 : 200, Thermo Fisher), and 100 ng/ml of DAPI (Solarbio) were used for immunofluorescence and applied for 10 min at room temperature. Images were processed with Nikon NID-elements software.

### 2.4. Immunohistochemistry

For immunohistochemistry, 5 *μ*m cryosections were collected on adhesive slides and fixed in 4% PFA for 10 min, then the slides were washed with PBS 3 times and then antigen-retrieved with sodium citrate using heating method with microwave. Slides were washed in PBS for 3 times and blocked in 5% BSA for 1 h at 37°C, then incubated at 4°C overnight with primary antibodies. The slides were stained following the SABC immunohistochemical kit (BOSTER) protocol, and nuclei were visualized by hematoxylin stain and observed using Nikon NID-elements software.

### 2.5. Western Blot Analysis

Protein isolation from tissue samples was performed with RIPA buffer (Bioss). Whole-cell lysates were subjected to SDS–PAGE. Protein expression was visualized using ultrasensitive ECL chemiluminescence detection kit (Absin) and quantified using a ChemiScope series Clinic Science Instruments (Clinx, China).

### 2.6. Histology

Histology was performed using Hematoxylin and Eosin and Masson's Trichrome staining according to the manufacturer's instructions (Solarbio).

### 2.7. qRT-PCR

Total RNA was isolated from TA muscle using TRIzol reagent (Invitrogen) following the manufacturer's instructions. cDNA was synthesized using a NovoScript SuperMix (NOVOProtein). Then, quantitative real-time PCR was performed using a PerfectStart Green qPCR SuperMix (Transgen) on a StepOnePlus Real-Time PCR Instrument (Themo Flsher Scientific). Beta-actin served as the internal control, and the relative express levels of mRNA were assessed through the comparative threshold cycle method (2^−ΔΔCt^) [15].

### 2.8. m^6^A MeRIP-Seq (Methylated RNA Immunoprecipitation and Sequencing)

The m^6^A MeRIP-Seq and data analyses were performed as previously described [16]. Briefly, Trizol reagent was used for the isolation of total RNA from muscle cells, and then, the PolyATtract mRNA Isolation System IV (Promega Z5310) was used to enrich mRNA from total RNA sample. 2 *μ*g of the purified mRNA was fragmentized with ZnCl^2^ buffer at 94°C for 5 min. The mRNA fragments were purified and then subjected to immunoprecipitation with anti-m^6^A antibody (1 : 100, Synaptic Systems, Cat No: 202003). After extensive wash, the methylated fragments were eluted by competition using free N6-Methyladenosine (Santa Cruz Biotechnology, sc-215524) and then used for library construction with the TruSeq Stranded mRNA Sample Prep Kits (Illumina RS-122-2101). The input and m^6^A MeRIP libraries were sequenced with Illumina NextSeq 500 with high output kit. The sequence reads mapping, peak calling and visualization, metagene analysis of m^6^A distribution, motif search, and gene ontology analysis were performed as previously described [17].

### 2.9. Polyribosome Bound mRNA-qPCR

The polyribosome-bound mRNA extraction was performed as described [18]. In brief, cells were pretreated with 100 *μ*g/ml cycloheximide for 3 min, followed by prechilled phosphate-buffered saline washes and addition of 2 ml cell lysis buffer (1% Triton X-100 in ribosome buffer (RB buffer), 20 mM HEPES-KOH (pH 7.4), 15 mM MgCl2, 200 mM KCl, 100 *μ*g/ml cycloheximide, and 2 mM dithiothreitol). After 30 min ice-bath, cell lysates were scraped and transferred to prechilled 1.5 ml tubes. Cell debris was removed by centrifuging at 16,200 g for 10 min at 4°C. Supernatants were transferred on the surface of 20 ml of sucrose buffer (30% sucrose in RB buffer). Polyribosomes were pelleted after ultracentrifugation at 185,000 g for 5 h at 4°C. Polyribosome-bound RNAs were isolated by using TRIzol RNA extraction reagent (Invitrogen), following the manufacturer's instructions. The subsequent treatment was the same as the qRT-PCR.

### 2.10. Cell Culture

The C2C12 mouse myoblast cells (Stem Cell Bank, Chinese Academy of Sciences, Shanghai, China) were cultured in Dulbecco's modified Eagle's medium (DMEM) high glucose (Gibco; Thermo Fisher Scientific, Inc., Waltham, MA, USA) supplemented with 15% fetal bovine serum (HyClone; GE Healthcare Life Sciences, Logan, UT, USA), 100 U/ml of penicillin, and 100 *μ*g/ml of streptomycin in 5% CO_2_ at 37°C. When the cells reached 80–90% confluence, they were differentiated by incubation in DMEM containing 2% horse serum (HyClone; GE Healthcare Life Sciences).

### 2.11. Statistical Analysis

Data are presented as the means ± standard deviation (S.D.) or standard error (S.E.). All of the statistical analyses were performed using Excel (Microsoft, Redmond, WA) or Prism (GraphPad Software Inc., La Jolla, CA). The two-tailed Student's *t*-test and one-way analysis of variance were used to calculate statistical significance. *p* value of <0.05 was considered significant.

## 3. Results

### 3.1. Knockout of METTL3 in MuSCs Inhibits Skeletal Muscle Regeneration

**Figure 1:**
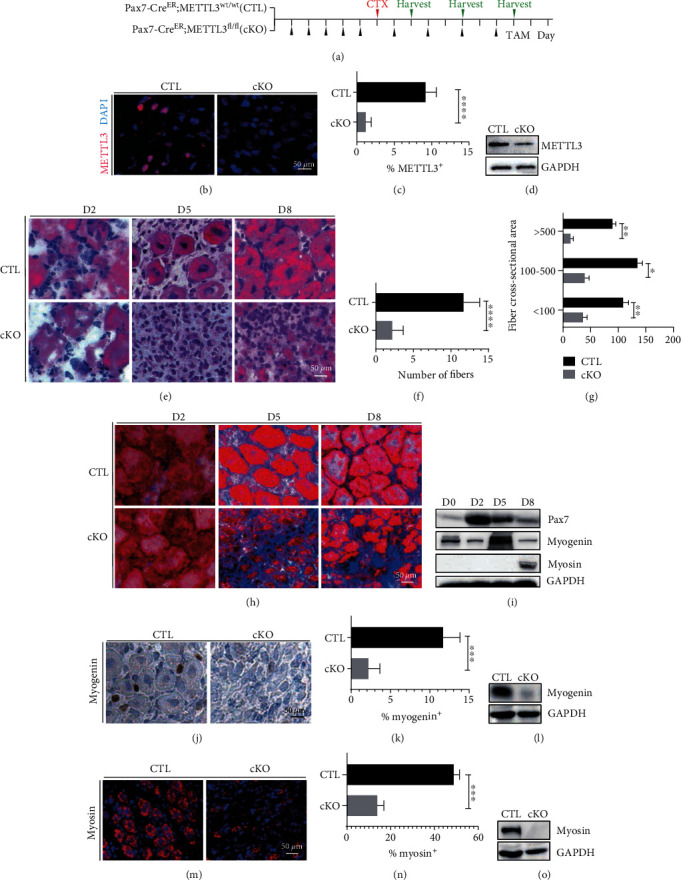
METTL3 is required for muscle regeneration *in vivo*. (a) Schematic outline of CTX injection in tamoxifen-treated CTL and cKO littermates. (b–d) Immunofluorescence staining and Western blot analysis of TA muscle after injection for 5 days for METTL3. (e) Hematoxylin and eosin (H&E) staining of TA muscles of METTL3 cKO mice and control littermates 2, 5, and 8 days after CTX injection. (f) Number of muscle fibers. (g) Area of regenerating centronuclear fibers. (h) Masson's Trichrome staining of TA muscles of METTL3 cKO and control littermates. (i) Western blot analysis of Pax7, myosin, myogenin, and METTL3 protein levels in control TA muscle at day 0, day 2, day 5, and day 8. (j) Immunofluorescence staining of TA muscles from METTL3 cKO and control mice detected by myogenin. (k) Quantifications of myogenin staining. (l) Western blot analysis of myogenin. (m) Immunohistochemical staining of myosin. (n) Quantifications of myosin staining. (o) Western blot analysis of myosin. *N* = 10. ^∗∗∗∗^*p* < 0.0001; ^∗∗∗^*p* < 0 .001; ^∗∗^*p* < 0.01; ^∗^*p* < .05. CTX: cardiotoxin; TAM: tamoxifen; TA: tibialis anterior; CTL: control; cKO: METTL3 conditional knockout.

In order to study the function of METTL3 in muscle stem cells and muscle regeneration, we established a genetic mouse model of conditional ablation of METTL3 in adult skeletal muscle stem cells. We used CRISPR/Cas9 technology to generate the METTL3 flox (METTL3^flox/wt^) mice, which was then crossed with Pax7CreERT2 mice to obtain conditional homozygous mice (Pax7CreERT2; METTL3 cKO^flox/flox^, cKO) and the corresponding control mice (Pax7CreERT2; METTL3^wt/wt^). After induction of METTL3 knockout in the muscle stem cells with tamoxifen (Figure 1(a)), the muscle regeneration was induced by injecting cardiotoxin (CTX) into the tibialis anterior (TA) muscles of METTL3 cKO mice and the control mice, and then, the muscle samples were collected at different timepoints after injection. Immunofluorescence analysis of METTL3 expression in TA muscles showed that METTL3 expression is depleted in mutant mice compared with control mice after CTX treatment (Figures 1(b) and 1(c)). Western blot analysis confirmed the effective depletion of METTL3 *in vivo* (Figure 1(d)). Regeneration efficiency was evaluated by observing fiber morphology (Figure 1(e)) sections 2, 5, and 8 days after CTX treatment. Our data showed that muscle regeneration was strongly retarded in the METTL3 cKO mice compared to the control (CTL) mice (Figure 1(f)). CSA (cross-sectional area) measurement showed that the most abundant fibers in METTL3 cKO muscle had a surface area smaller than 100 *μ*m^2^, whereas larger fibers were significantly less represented than in control muscle (Figure 1(g)). Decreased regenerative capacity was also observed by Massion staining, since at day 5 and 8 post-CTX treatment METTL3-ablated muscles displayed increased fibrosis and fewer regenerating fibers (Figure 1(h)).

We next determined the function of METTL3 in muscle regeneration by evaluating the expression of key myogenic factors. Many protein markers participate in the muscle injury repair process, among which Pax7 [19] is the MuSCs proliferation marker, myogenin, the early differentiation marker, and myosin, the late differentiation marker. Our data showed that Pax7 expression was increased on day 2 after injury because MuSCs needed to proliferate and then participate in the repair; myogenin was increased on day 5, while myosin was increased on day 8 post-CTX treatment (Figure 1(i)). Our data displayed a noticeable decrease of myogenin expression in METTL3 cKO muscle tissues relative to the normal control (Figures 1(j)–1(l)). Not surprisingly, myosin expression was also significantly reduced in METTL3 cKO mice (Figures 1(m)–1(o)). Taken together, our data uncovered the important physiological function of METTL3 in regulation of muscle regeneration *in vivo*.

### 3.2. METTL3 Affects Muscle Regeneration by Regulating the Proliferation of MuSCs

**Figure 2:**
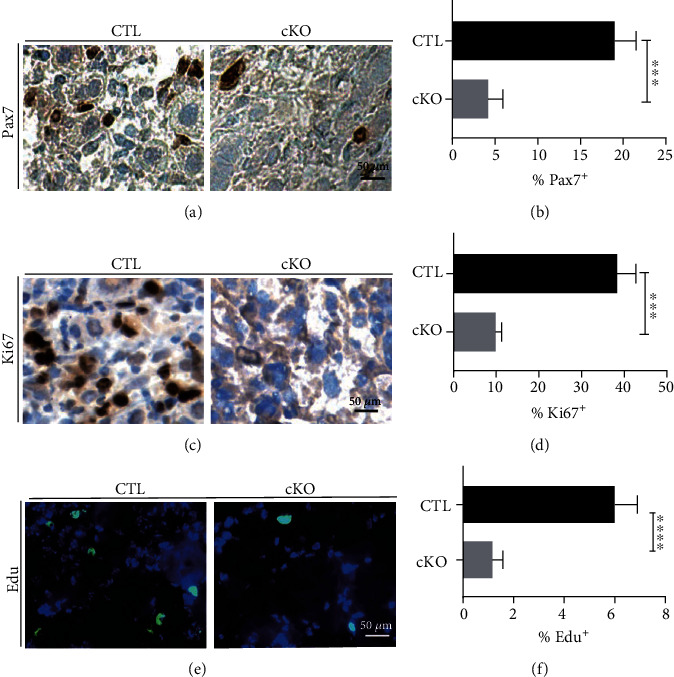
METTL3 regulates muscle stem cell proliferation *in vivo*. (a–d) Immunohistochemical analysis of TA muscle samples from METTL3 cKO and control by Pax7 and Ki67 antibodies, respectively. Quantifications of Pax7 and Ki67 staining were shown on the right. (e) EdU of TA muscles and (f) images of cell numbers 5 days after isolation from injured muscles of METTL3cKO and control mice. ^∗∗∗∗^*p* < 0.0001; ^∗∗∗^*p* < 0.001. CTL: control; cKO: METTL3 conditional knockout.

Next, we evaluated the function of METTL3 in regulation of muscle stem cells. Our immunohistochemical staining showed that knockout of METTL3 resulted in significant decrease of Pax7-expressing (Pax7^+^) cells in TA muscles after CTX injury (Figures 2(a) and 2(b)). Pax7^+^ cells are the muscle stem cells required for the repair of damaged muscle tissue after injury [20]. Our results demonstrated that METTL3 knockout leads to a decrease in Pax7+ MuSCs, which therefore leads to impaired repair of muscle injury. We also assessed cell proliferation by immunostaining for Ki67 in the injected TA muscle harvested from mice mentioned above (Figures 2(c) and 2(d)), which further confirmed that METTL3 knockout leads to reduced proliferation of MuSCs, which in turn affects damage repair. 5-Ethynyl-2′-deoxyuridine (EdU) is a thymidine analog that can be incorporated into replicating DNA for detection of cell proliferation. Similarly, we found that METTL3 knockout led to a decrease proliferation in MuSCs indicated by reduced EdU incorporation (Figures 2(e) and 2(f)). Overall, our data revealed that METTL3 affects muscle regeneration by regulating the proliferation of MuSCs.

### 3.3. Conditional Knockin of METTL3 in Mouse Stem Cells Promotes Muscle Regeneration

**Figure 3:**
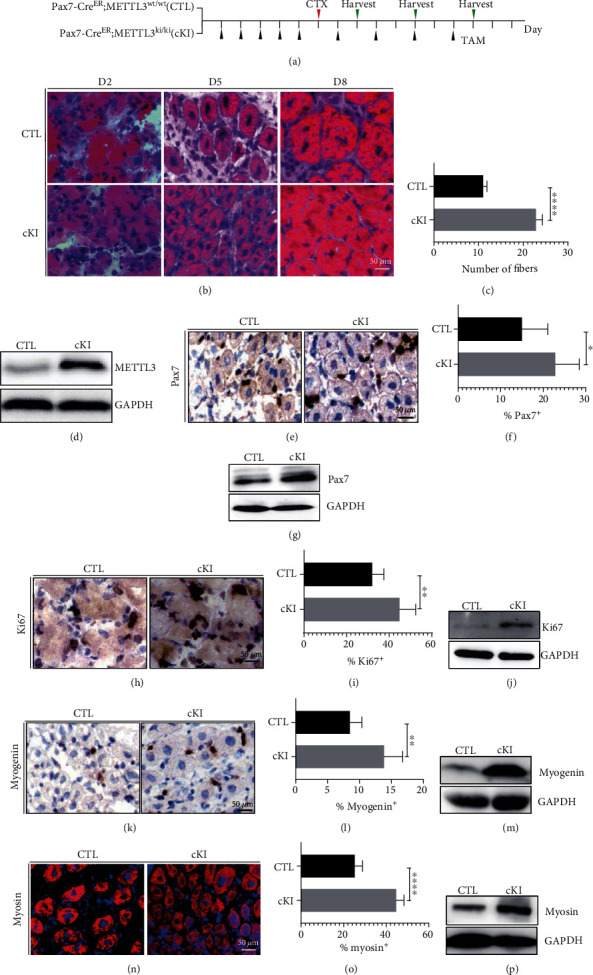
METTL3 conditional knockin promotes muscle regeneration *in vivo*. (a) Schematic outline of CTX injection in TAM-treated control and METTL3 cKI littermates at the age of 8–9 weeks. (b) H&E staining of representative tibialis muscle sections from METTL3 cKI, control mice treated 2, 5, and 8 days after CTX injection. (c) Quantified of muscle fibers. (d) The upregulation effect of METTL3 was verified at protein levels by Western blot. (e, h, k) Immunohistochemical and (n) immunofluorescence staining of TA muscle from METTL3 cKI and control detected by Pax7, Ki67, myogenin, and myosin antibodies. (f, i, l, o) Quantifications of Pax7+, Ki67+, myogenin+, and myosin+ cells were shown on the right. (g, j, m, p) Western blot detected the protein expression levels of Pax7+, Ki67+, myogenin, and myosin. Data represent mean ± SEM. *N* = 5. ^∗∗∗∗^*p* < 0.0001, ^∗∗^*p* < 0.01, ^∗^*p* < 0.05. CTX: cardiotoxin; TAM: tamoxifen; TA: tibialis anterior; CTL: control; cKI: METTL3 conditional knockin.

To further assess potential roles of METTL3 in skeletal muscle regeneration, we developed muscle stem cell-specific METTL3 conditional knockin mouse (Pax7CreERT2; METTL3^ki/wt^, cKI). After induction of METTL3 knockin in muscle stem cells with tamoxifen, the cKI and control mice were subjected to CTX treatment to induce muscle regeneration (Figure 3(a)). At day 5 post-CTX treatment, we observed a higher number of regenerating centronuclear fibers in METTL3 cKI mice compared to that in the control mice (Figures 3(b) and 3(c)). Western blot analysis showed the successful overexpression of METTL1 in the cKI mice (Figure 3(d)). This indicates knockin of METTL3 promotes the *in vivo* muscle regeneration upon injury. To confirm that an increased number of MuSCs is responsible for faster muscle regeneration *in vivo*, we assessed the muscle stem cell marker and cell proliferation by immunostaining for Pax7 and Ki67 (Figures 3(e)–3(j)). The proliferation index clearly showed that the overexpression of METTL3 could cause the increase of Pax7+ MuSCs, which then promotes muscle regeneration. Moreover, detection of myogenin and myosin expression further supported that METTL3 knockin promotes the muscle regeneration *in vivo* (Figures 3(k)–3(p)). Taken together, our muscle regeneration models using the METTL3 cKO and cKI mice strongly supported the critical physiological function of METTL3 in regulation of muscle stem cells and muscle regeneration.

### 3.4. METTL3 Regulates m^6^A Modification of Notch Signaling Pathway Components

**Figure 4:**
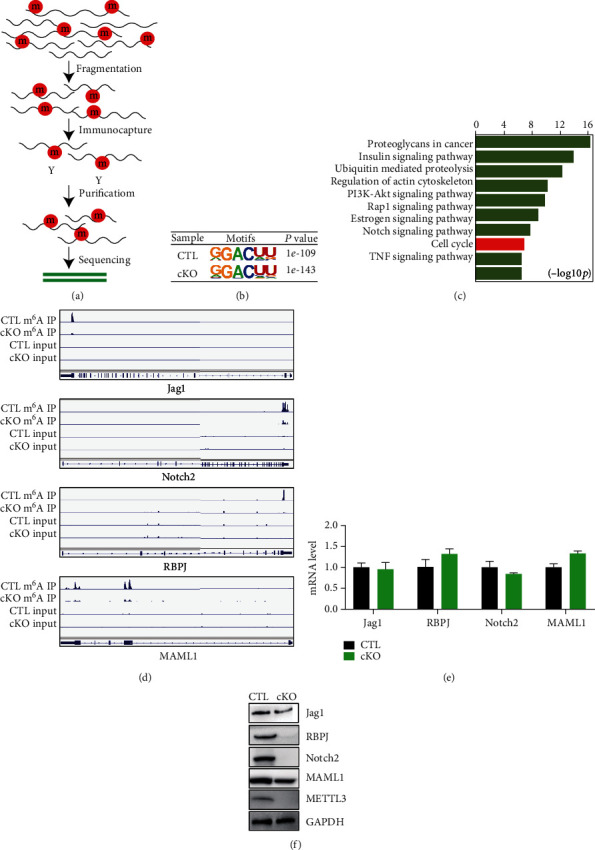
METTL3 regulates m^6^A modification of Notch signaling pathway components. (a) Schematic representation of m^6^A MeRIP-Seq. (b) m^6^A motif identified in the MeRIP-Seq. (c) Enriched KEGG molecular function pathways identified from differentially m^6^A-enriched genes in METTL3 cKO vs. CTL D2 samples. (d) Notch signaling pathway identified in the MeRIP-Seq. (e, f) qRT-PCR and Western blot analyzed of Notch signaling pathway. CTL: control; cKO: METTL3 conditional knockout.

To dissect the underlying mechanism of METTL3-mediated m^6^A modification in regulation of MuSC fate and muscle regeneration, we performed the m^6^A MeRIP-Seq (Figure 4(a)) to identify the critical m^6^A targets in MuSCs. Our result identified the consensus GGAC m^6^A motif in the m^6^A MeRIP-Seq data (Figure 4(b)). Gene ontology analysis of the differentially methylated mRNAs between the METTL3 cKO and control samples revealed that METTL3 regulates the m^6^A modification of several important signaling pathways (Figure 4(c)). Of which, the Notch signaling pathway plays important functions in regulation of muscle stem cells and muscle regeneration. Our m^6^A MeRIP-Seq showed that the peak value of m^6^A in Notch receptor (Notch2), Notch ligand (Jag1), transcription factor (RBPJ), and activators (MAML1) in METTL3 cKO group was significantly decreased in the METTL3 cKO sample (Figure 4(d)). This result directly indicates that METTL3 affects the m^6^A modification level of Notch signaling pathway components in the regenerating muscle.

Next, Western blot and qRT-PCR were used to determine the expression of Notch signaling pathway components in the muscle samples of METTL3 cKO and control mice. Compared with the control group, Western blot results showed that the protein expression level of Notch signaling pathway components in the muscle of METTL3 cKO group was significantly reduced, while qRT-PCR results showed no significant changes in their mRNA levels (Figures 4(e) and 4(f)). This suggests that METTL3 affects muscle regeneration by affecting the m^6^A methylation level of Notch signaling pathway at translation level.

### 3.5. METTL3 Regulates the Differentiation of C2C12 Myoblast Cells

**Figure 5:**
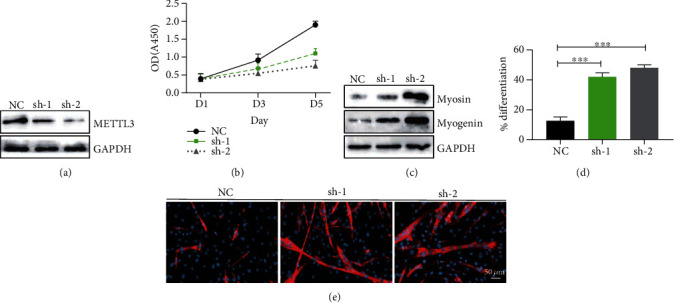
Deletion of METTL3 affects C2C12 myoblast progenitor cell proliferation and differentiation. (a) Western blot detection of METTL3 expression. (b) Proliferation ability significantly decreased after METTL3 knockout determined by cell counting Kit-8 at indicated days. (c) Western blot analysis of the expression of early differentiation marker myogenin and late differentiation marker myosin in C2C12 cells. (d) Myosin staining (red) was performed 3 days later using an anti-myosin antibody, and the nuclei were counterstained with DAPI. (e) Differentiation index in the METTL3-depleted and control cells. Data represent mean ± SEM. ^∗∗∗^*p* < 0.001. NC: negative control; sh-1: METTL3 shRNA-1; sh-2: METTL3 shRNA-2.

We have demonstrated that METTL3 affects muscle regeneration by affecting the m^6^A methylation level of Notch signaling pathway *in vivo*. To further study the function and mechanisms of METTL3 in regulation of downstream target expression, we knocked down METTL3 expression in C2C12 myoblast progenitor cells (Figure 5(a)). We then examined the proliferation and differentiation ability of the myoblasts after METTL3 knockdown. The cell proliferation results revealed that the proliferation ability of C2C12 myoblasts was significantly reduced after METTL3 knockdown (Figure 5(b)). In addition, downregulation of METTL3 promoted the ability of C2C12 cells to differentiate into muscle fibers detected by Western blot (Figure 5(c)). Compared with the NC group, in shMETTL3 cells, both the early differentiation marker myogenin and the late differentiation marker myosin are upregulated. Meanwhile, we performed myosin staining to visualize the differentiated myofibers and found that the differentiated myofibers are significantly increased in the METTL3 knockdown C2C12 myoblasts (Figures 5(d) and 5(e)). These data suggest that METTL3 depletion in C2C12 cells results in decreased proliferation and accelerated differentiation of the myoblast progenitor cells.

### 3.6. METTL3-Mediated m^6^A Modification Regulates Notch Signaling Pathway at Translation Level

**Figure 6:**
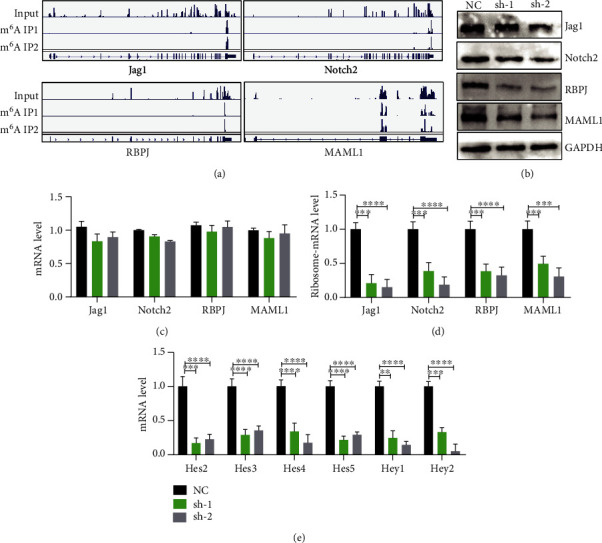
METTL3 regulates the mRNA translation efficiency of Notch signaling pathway components. (a) The Notch pathway genes with m^6^A modification. (b) The protein levels of Notch signaling pathway in the METTL3-depleted and control cells. (c) mRNA expression of Notch signaling pathway analyzed by q-PCR. (d) Polyribosome-bound mRNA-qPCR was used to detect translation efficiency. (e) The downstream gene expression of Notch signaling pathway was detected by qRT-PCR. ^∗∗∗∗^*p* < 0.0001; ^∗∗∗^*p* < 0.001; ^∗∗^*p* < 0.01. NC: negative control; sh-1: METTL3 shRNA-1; sh-2: METTL3 shRNA-2.

In order to study the mechanisms of METTL3 in regulation of proliferation and differentiation in C2C12 myoblast cells, we conducted m^6^A MeRIP-Seq sequencing analysis (Figure 6(a)). Similar to the *in vivo* result, our sequencing results showed strong m^6^A modification on the mRNAs of Notch signaling pathway components (Jag1, Notch2, RBPJ, and MAML1) in myoblasts. Western blot results showed that the protein expression levels of Jag1, Notch2, RBPJ, and MAML1 decreased after METTL3 knockdown (Figure 6(b)). However, there was no significant difference in their mRNA levels (Figure 6(c)). To confirm our findings, we used polyribosome-associated mRNA-qPCR to detect the translation efficiency of Notch signaling pathway components. The results showed the mRNA translation efficiencies of Notch signaling pathway components are significantly reduced (Figure 6(d)), which directly leads to the decrease of protein expression level. At the same time, the expression levels of target genes (Hes2, Hes3, Hes4, Hes5, Hey1, and Hey2) in Notch signaling pathway were also significantly downregulated, further confirming that the depletion of METTL3 inhibits the Notch signaling pathway (Figure 6(e)). Overall, METTL3 depletion regulates the proliferation and differentiation of C2C12 cell by Notch signaling pathway at translation level.

### 3.7. YTHDF1 Regulates Translation of Notch Signaling Pathway Components

**Figure 7:**
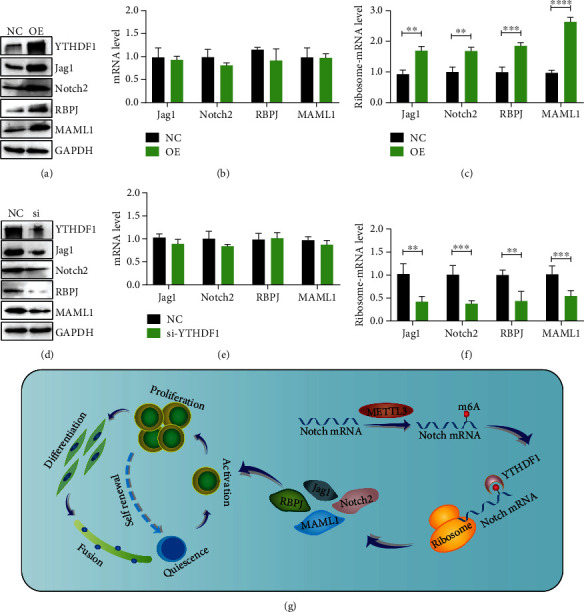
YTHDF1 regulates the mRNA translation efficiency of Notch signaling pathway components. (a) The protein levels of Notch signaling pathway in the METTL3 overexpressed or (d) depleted and control cells. (b, e) mRNA expression of Notch signaling pathway analyzed by q-PCR. (c, f) Polyribosome-bound mRNA-qPCR was used to detect translation efficiency. (g) Working model of METTL3 regulates muscle stem cells by Notch signaling pathway. ^∗∗∗∗^*p* < 0.0001; ^∗∗∗^*p* < 0.001; ^∗∗^*p* < 0.01. NC: negative control; OE: YTHDF1 overexpressed; si: YTHDF1 siRNA.

Given that METTL3 knockdown decreases the translation of Notch pathway components and the m^6^A reader protein YTHDF1 functions in promoting the translation of m^6^A modified mRNAs [8], we therefore investigated the potential function of YTHDF1 in regulation of Notch signaling pathway. We first overexpressed YTHDF1 in C2C12 myoblast progenitor cells (Figure 7(a)) and then examined the mRNA levels and the translation efficiency of Notch signaling pathway components. The results showed there was no significant difference in their mRNA levels after YTHDF1 overexpression (Figure 7(b)). However, the mRNA translation efficiencies of Notch signaling pathway components are significantly increased (Figure 7(c)). On the other hand, knockdown of YTHDF1 decreased the translation of Notch pathway mRNAs without changing their mRNA levels (Figures 7(d)–7(f)). Overall, our data revealed that the METTL3-m6A-YTHDF1 axis regulates the Notch signaling pathway and controls muscle stem cells and muscle regeneration (Figure 7(g)).

## 4. Discussion

This study reported that METTL3-mediated m^6^A modification regulates the muscle stem cells and muscle regeneration by affecting the translation of Notch signaling pathway. Emerging data revealed that METTL3-mediated m^6^A represents a ubiquitous modification in RNA of all higher eukaryotes that can affect mRNA metabolism, maintain cell self-renewal, and control the embryonic differentiation [21–23]. In our study, we showed that muscle stem cell-specific METTL3 knockout results in decreased proliferation activity of muscle stem cells and slower repair of muscle damage, whereas conditional knockin of METTL3 can promote muscle regeneration *in vivo*. Mechanistically, using generating muscle samples and myoblasts, we also found that METTL3 regulates MuSC regeneration through regulating the m^6^A modification of Notch signaling pathway at translation level.

There are two key events in the *in vivo* muscle regeneration model: after injury, the muscle stem cells are first activated and then undergo extensive proliferation to expand the stem cell population; after that, the stem cells undergo differentiation to reconstitute the muscle structure. Our data showed that METTL3 cKO in the muscle stem cells decreases proliferation of muscle stem cells, which leads to insufficient number of muscle stem cells that can be used for injury repair, eventually results in the impaired muscle reconstitution, as showed by the reduced level of myogenin and myosin. On the other hand, in the *in vitro* myoblast model, our data showed that knockdown of METTL3 decreases myoblast proliferation and results in the predifferentiation of myoblasts, as reflected by the increased expression of myogenin and myosin after induction of differentiation. Therefore, both our *in vivo* and *in vitro* data supported the essential function of METTL3-mediated m^6^A modification in regulation of muscle stem cells and muscle regeneration.

The Notch signaling pathway plays important functions in regulation of muscle stem cells and muscle regeneration. Canonical Notch signaling is initiated by interaction of the extracellular domain of ligands (DLL-1, -3, -4 and Jag-1 and -2) with their counterparts on one of the four receptors (Notch1–4), then the Notch receptors are cleaved and releases into the nucleus to bind to the transcription factor RBPJ and recruit coactivators including MAML1 to activation target gene translation. Notch signaling pathway is required for MuSC maintenance, activation, proliferation, and differentiation and muscle regeneration [24–30]. The genes of the Notch signaling pathway, such as Notch2, Jag1, RBPJ, and MAML1, which are involved in the control of the stem cell's fate and behavior, are essential for the myogenic progress [31, 32]. In our study, it was found that METTL3 knockout led to reduction of m^6^A modification levels of Notch family genes. And upon METTL3 knockout, the mRNA level of Notch signaling pathway did not change, but the protein expression significantly decreased. We further revealed that depletion of METTL3 resulted in decreased mRNA translation efficiencies of the Notch pathway components. And the m^6^A reader protein YTHDF1 promotes the mRNA translation of Notch pathway components. These data revealed that the METTL3-mediated m^6^A modification facilitates muscle stem cells and muscle regeneration through regulation of Notch signaling pathway.

Taken together, our findings provide a new insight in the pivotal regulatory role of METTL3-mediated m^6^A modification in muscle stem cells and muscle regeneration. Our data uncovered the novel posttranscriptional mechanisms underlying muscle stem cell fate and muscle regeneration and provided molecular basis for the development of therapeutic strategies for muscle-related diseases including muscle injury and muscular dystrophies.

## Data Availability

The sequencing data were deposited into Gene Expression Omnibus (GSE169432).
